# Inhibition of colony-stimulating factor 1 receptor early in disease ameliorates motor deficits in SCA1 mice

**DOI:** 10.1186/s12974-017-0880-z

**Published:** 2017-05-25

**Authors:** Wenhui Qu, Andrea Johnson, Joo Hyun Kim, Abigail Lukowicz, Daniel Svedberg, Marija Cvetanovic

**Affiliations:** Department of Neuroscience, Institute for Translational Neuroscience, University of Minnesota, 2101 6th Street SE, Minneapolis, MN 55455 USA

**Keywords:** Spinocerebellar Ataxia type 1, Neuroinflammation, Cerebellum, Motor deficit, ATAXIN-1, Microglia, Purkinje neurons, Glia, SCA1

## Abstract

**Background:**

Polyglutamine (polyQ) expansion in the protein Ataxin-1 (ATXN1) causes spinocerebellar ataxia type 1 (SCA1), a fatal dominantly inherited neurodegenerative disease characterized by motor deficits, cerebellar neurodegeneration, and gliosis. Currently, there are no treatments available to delay or ameliorate SCA1. We have examined the effect of depleting microglia during the early stage of disease by using PLX, an inhibitor of colony-stimulating factor 1 receptor (CSFR1), on disease severity in a mouse model of SCA1.

**Methods:**

Transgenic mouse model of SCA1, *ATXN1[82Q]* mice, and wild-type littermate controls were treated with PLX from 3 weeks of age. The effects of PLX on microglial density, astrogliosis, motor behavior, atrophy, and gene expression of Purkinje neurons were examined at 3 months of age.

**Results:**

PLX treatment resulted in the elimination of 70–80% of microglia from the cerebellum of both wild-type and *ATXN1[82Q]* mice. Importantly, PLX ameliorated motor deficits in SCA1 mice. While we have not observed significant improvement in the atrophy or disease-associated gene expression changes in Purkinje neurons upon PLX treatment, we have detected reduced expression of pro-inflammatory cytokine tumor necrosis factor alpha (TNFα) and increase in the protein levels of wild-type ataxin-1 and post-synaptic density protein 95 (PSD95) that may help improve PN function.

**Conclusions:**

A decrease in the number of microglia during an early stage of disease resulted in the amelioration of motor deficits in SCA1 mice.

**Electronic supplementary material:**

The online version of this article (doi:10.1186/s12974-017-0880-z) contains supplementary material, which is available to authorized users.

## Background

Neuroinflammation, the brain’s response to injury, is associated with many neurodegenerative diseases, including Alzheimer disease (AD), Parkinson disease (PD), amyotrophic lateral sclerosis (ALS), multiple sclerosis (MS), Huntington disease (HD), and spinocerebellar ataxia type 1 (SCA1) [1–7]. Emerging evidence suggests that neuroinflammation can actively contribute to disease pathogenesis [8–11], but its role in disease is complex and includes both amelioration and promotion of disease progression. While different brain cell types contribute to neuroinflammation, microglia, the resident immune cells of the brain, are considered to be the primary mediators of neuroinflammation [12].

During brain development, microglia are responsible for the removal of synapses and apoptotic cells [13–16]. In the healthy adult brain, microglia’s long processes actively sample brain environment for neuronal activity and signs of brain injury [17]. In a reaction to brain injury, microglia undergo morphological and functional changes, including enlargement of cell bodies and thickening of their processes and increased expression of pro-inflammatory cytokines. It has been proposed that the extent of microglial activation and thereby their contribution to the pathogenesis depends on the type and extent of injury [5]. For example, many studies have found chronically activated microglia to be harmful and worsen the disease outcome in ALS, HD, PD, and AD [8, 18–23]. However, there are also studies that suggest activated microglia may be beneficial in these diseases [24–31].

Spinocerebellar ataxia type 1 (SCA1) is a dominantly inherited neurodegenerative disorder characterized by progressive loss of motor coordination, cognitive impairments, and depression [32, 33]. SCA1 is caused by an expansion of glutamine-encoding CAG (Q) repeats in the gene *Ataxin-1* (*ATXN1*) [34, 35]. There are eight other neurological diseases, including HD, that share the polyQ mutational mechanism [33]. One of the most intriguing characteristics of polyQ diseases is their limited pathology despite the widespread expression of disease-causing proteins. While ATXN1 is ubiquitously expressed, Purkinje neurons of the cerebellum are the most affected cells in SCA1 [36]. For this reason, most studies so far investigated Purkinje neuron-intrinsic pathology in SCA1. Recent evidence suggested that glial cells might also play a role in the pathogenesis of SCA1. Proton magnetic resonance spectroscopy studies suggested that gliosis correlates with disease severity in SCA1 patients, and we have recently demonstrated that microglia are activated early in several mouse models of SCA1 [4, 37]. However, whether and how microglia contribute to SCA1 is unknown. To study the role of microglia in SCA1, we reduced their numbers by using the pharmacological inhibition of colony-stimulating factor 1 receptor (CSF1R) signaling, which is essential for microglial survival. For this, we used PLX3397, inhibitor of CSF1R, referred to as “PLX” (Plexxicon Inc.), that has been shown to cause microglial depletion within several days of administration [38]. PLX also inhibits c-kit and Flt3 pathways, but few studies have shown their participation in SCA1. Mice treated with PLX for 2 months did not demonstrate any motor deficits or other abnormality, suggesting that long-term PLX administration is safe. We have focused our study on the early stages of SCA1 for several reasons. First, microglia are activated early in SCA1 mice. Second, studies in several neurodegenerative diseases, including SCA1, suggested that it is difficult to ameliorate disease pathology during the late stages after neuronal loss had already occurred [39]. Third, because SCA1 is an inherited disease, starting the treatment early is feasible. Fourth, microglia have recently been implicated in the early synaptic loss in Alzheimer disease; since loss of Purkinje cell synapses starts early in SCA1 as well, we reasoned that depleting microglia may ameliorate synaptic loss in SCA1 [8, 40, 41].

## Methods

### Compounds

PLX3397 was provided by Plexxicon Inc. From weaning, mice were fed chow with PLX3397 (Plexxicon, Inc.) to achieve approximate concentration of 200 mg/kg of mouse body weight.

### Mouse lines

*ATXN1[82Q]* mice were generated as previously described [42]. Originally generated on a C57BL/6 J–129/SvEv mixed background, these mice were backcrossed for more than ten generations with FVB mice to avoid confounding effects of genetic background. Because our previous studies have detected no gender-specific effects, we have used an equal mix of animals of both sexes for our experiments. All animal experiments were performed in compliance with the National Institutes of Health’s Guide for the Care and Use of Laboratory Animals and the University of Minnesota Institutional Animal Care and Use Committee. From weaning, 10 *Atxn1[82Q]* and 16 wild-type littermates were fed control chow, and 12 *Atxn1[82Q]* and 12 wild-type littermates were fed chow with PLX3397 (Plexxicon, Inc.) to achieve approximate concentration of 200 mg/kg of mouse body weight, for the next 2 months when mice were analyzed for disease severity.

### Rotating rod and balance beam tests

*ATXN1[82Q]* mice and their wild-type littermates treated with PLX or control chow were sequentially assayed at balance beam and rotarod at the age of 3 months. All experiments were performed blinded with respect to the knowledge of genotype or treatment.

The rotarod test was performed as previously described [43]. Briefly, mice were placed on the rotarod apparatus (Ugo Basile) that accelerates from a speed of 4 rotations per minute (rpm) to 40 rpm over a 5-min period. The time it takes for a mouse to fall off the rotarod is recorded to a maximum of 10 min. Mice were subjected to four trials per day for four consecutive days, with at least 10 min of rest between each trial. Data for the performance on day 4 was analyzed using one-way ANOVA with pairwise permutation tests, and Benjamini-Hochberg procedure *p* value adjustment, in R with “coin” package [44] and “rcompanion” package [45].

The balance beam tests a mouse’s ability to maintain balance while traversing a meter-long narrow beam to reach a dark goal box. Recorded measurement is the time taken to cross the square beam (latency) and the numbers of paw slips. Ataxic mice have a longer latency and more paw slips than wild-type littermates; however, when we examined them at 3 months of age, SCA1 mice had only longer latency. Data were statistically analyzed using ANOVA with Bonferroni post hoc test. Significance was assumed at *p* < 0.05.

### Immunohistochemistry

Mouse brains were fixed overnight in 4% formaldehyde, incubated in 30% sucrose, then sectioned on cryostat (Leica, CM 1850) into 45-μm sections. Sections were washed three times in cold phosphate buffered saline (PBS) and incubated in blocking buffer (3% normal donkey serum in PBST (1% Triton X-100 phosphate buffered saline (PBS)) for 1 h at room temperature (RT), followed by an overnight incubation at 4 °C in blocking buffer containing the relevant primary antibody [anti-GFAP (Z0334, DAKO), anti-Iba1 (019–19741, WAKO), anti-Calbindin-D-28K (C9848, Sigma Aldrich), anti-VGLUT2 (MAB5504, Millipore), anti-ataxin1 (11NQ, a gift from Dr. Harry Orr)]. Samples were washed three times with PBS and incubated overnight at 4 °C with the relevant fluorescently labeled secondary antibody. Stained sections were mounted on slides with Vectashield mounting media containing DAPI (Vector Laboratories) for observation under the confocal microscope (Olympus FV1000). For each mouse, we imaged at least six different, randomly selected cerebellar lobules. Each image consisted of 20-μm Z-stacks, composed of 1 μm thick image slices. Images were analyzed using FIJI (ImageJ) software. For calbindin staining, we drew a straight line extending from the Purkinje cell body to the end of the molecular layer in at least two places for each lobule. We then used the Measure function in ImageJ to quantify the length of the line to obtain measurement of the width of the molecular layer and the average intensity along the line to obtain calbindin intensity. To assess the extension of climbing fiber synapses along the Purkinje neuron dendrite, the distance from the Purkinje neuron cell bodies to the end of VGLUT2 staining was measured and divided by width of the molecular layer (measured using calbindin staining as described above). For GFAP staining, we used the Measure function in ImageJ to quantify the intensity of signal in the molecular layer that was then normalized to the average intensity in the control group (wild-type mice). To quantify the number of microglia, we divided the number of Iba1 positive cells by the area in which they were counted, to obtain their density. Staining, microscopy, and image analysis were performed blinded to the experimental groups. Data were analyzed using ANOVA with Bonferroni post hoc test using GraphPad Prism software (GraphPad software).

### Quantitative real time RT-PCR

Total RNA was isolated from the cerebella using TRIzol (Invitrogen) and treated with DNAse (TURBO Dnase, Thermo Fisher Scientific). Complementary DNA (cDNA) synthesis was performed in duplicate, using Superscript© III First-Strand Synthesis SuperMix (Invitrogen) and random hexamer primers. The expression level of each gene was determined on a Light Cycler 480 II (Roche) using Light Cycler 480 SYBR Green PCR I Master mix (Roche). Cycling conditions were 5 min at 95 °C, followed by 40 cycles of 95 °C for 15 s and 56 °C for 1 min. Samples were analyzed in triplicate, and a melting curve analysis was performed in each sample at the end of the qPCR reaction. The Itgam, Trem2, and Aif1 primers used were PrimeTime qPCR Primers (IDT). Expression levels of mouse 18S RNA (forward primer: 5′ AGT CCC TGC CCT TTG TAC ACA 3′ and reverse primer: 5′ CGA TCC GAG GGC CTC ACT A-3′) were used as internal controls. Primers used for the Magenta cluster genes (*calbindin, ITPR, INPP5a, Garnl3, Pcp4, Homer3,* and *Rgs8*) were from Ingram et al. [46], and primers used for astroglial genes *Kir4.1, EAAT1, P2RY, Aqp4, C3, S100,* and *Glul* were PrimeTime qPCR primers (IDT). Relative gene expression was determined by the 2^−ΔΔCt^ method [47]. The threshold cycle (Ct) value was determined for target genes and the endogenous internal controls in each sample. The difference between target gene and 18S RNA control Ct values was determined for each sample, resulting in the ΔCt value. The ΔCt of a calibrator, a wild-type or control *ATXN1[82Q]* sample, was subtracted from each sample ΔCt to yield the ΔΔCt value. Relative fold change was calculated as 2^−ΔΔCt^. Data was analyzed using ANOVA with Bonferroni post hoc test using GraphPad Prism software (GraphPad software).

### Western blotting

The cerebella were dissected from mice and lysed in RIPA lysis buffer (50 mM Tris HCl, pH 7.4, 150 mM NaCl, 1% sodium deoxycholate, 1% NP-40, 0.2% SDS, phosphatase (Sigma) and protease inhibitors cocktail (Roche)). After three cycles of freeze and thaw, proteins were separated on a 12% or 15% SDS-PAGE gel and transferred onto a nitrocellulose membrane. The following primary antibodies were used: anti-ATXN1 (rabbit 11NQ, Orr lab), anti-Iba1 (DAKO), anti-synapsin (Synaptic systems), anti-PSD95 (Biolegend), anti-TNF alpha (Abcam ab34674), and alpha-tubulin (mouse, Sigma). Signals from secondary antibodies linked to horseradish peroxidase (HRP) (GE Healthcare) were detected using Amersham ECL Western Blotting Detection Reagent (GE Healthcare) and ImageQuant LAS 4000 imager (GE Healthcare); protein levels were quantified using ImageQuant (GE healthcare) and ImageJ software. For tumor necrosis factor alpha (TNFα), we quantified the 20 kD band, and for synapsin, we quantified synapsin 2a band. Data was analyzed with one-way ANOVA followed by Bonferroni post hoc test and Student’s *t* test with Welch’s correction.

### Statistical analysis

Statistical tests were performed with GraphPad Prism or R. For rotarod, we have used one-way ANOVA followed by pairwise permutation tests, and Benjamini-Hochberg procedure *p* value adjustment, in R with “coin” package [44] and “rcompanion” package [45]. For balance beam test, we used one-way ANOVA followed by Bonferroni post hoc test. For IHC, qRT-PCR, and Western blot quantification, we used one-way ANOVA followed by Bonferroni post hoc test. For quantifying mutant ataxin-1[82Q] expression, since we compared just two groups, we used Student’s *t* test with Welch’s correction.

## Results

### PLX treatment reduces microglia in the cerebella of *ATXN1[82Q]* mice

We have previously demonstrated an increase in the density of microglia, microglial hypertrophy, and altered expression of pro-inflammatory genes during the early stages of disease in several SCA1 mouse lines [4]. We then wanted to test if this early activation of microglia contributes to the onset or severity of SCA1. To test if microglia play a role during the early stage, we depleted microglia in SCA1 mice at weaning using a pharmacological approach. PLX is an inhibitor of CSF1R signaling, which is required for the maintenance of microglia in the brain [38]. Mice were treated with PLX added to the mouse chow (at a concentration of 200 mg/kg of mouse body weight), starting at 3 weeks of age until 3 months of age. Control wild-type and *ATXN1[82Q]* mice were fed regular chow without drug and served as the baseline for behavior and pathology analysis.

**Fig. 1:**
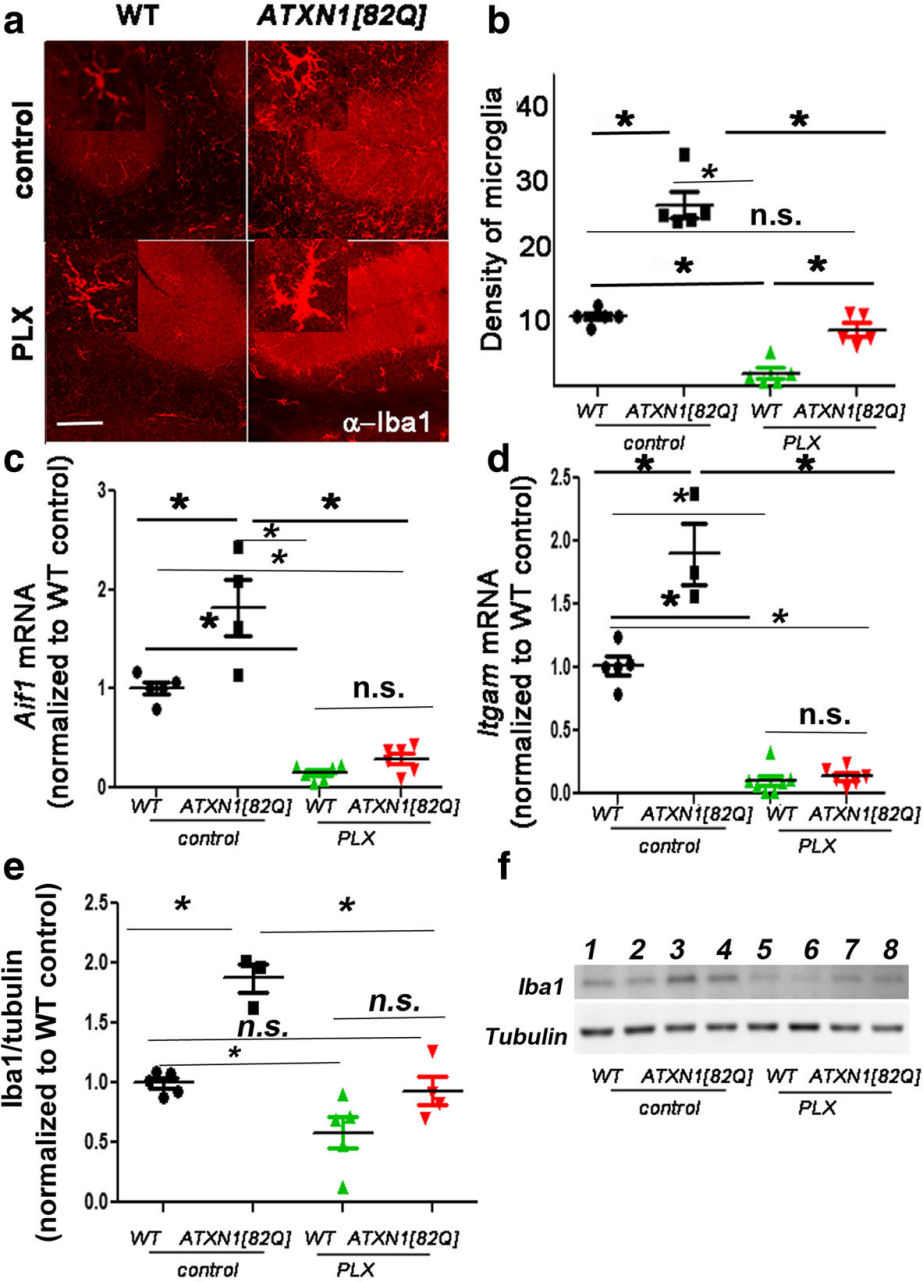
PLX treatment depletes microglia in the cerebella of *ATXN1[82Q]* mice. *ATXN1[82Q]* and wild-type (WT) mice were fed PLX3397(PLX) or regular (control) chow from 3 weeks of age. At 3 months of age, the cerebella was dissected and used for immunofluorescence (IFC) or RNA analysis. **a** Representative confocal images of 3-month-old mice stained with a microglia specific Iba-1-antibody. *Insets* in each image demonstrate hypermorphy of PLX-treated surviving microglia. *Scale bar*, 100 μm. **b** Density of Iba1 positive microglia in the cerebellar cortex (*N* = 5 per each genotype). **c**, **d** Cerebellar RNA was used for quantitative RT-PCR with primers for microglia specific genes *Aif1*(**c**, *N* ≥ 4) and *Itgam* (**d**, *N* ≥ 3). **e**, **f** Western blotting for Iba-1 demonstrates decrease in PLX-treated mice. (**f**, *N* ≥ 3). Each dot represents one mouse, and values indicate mean ± SEM. *Asterisk* indicates *p* < 0.05 by one-way ANOVA followed by Bonferroni post hoc test, and *n.s*. indicates that results are not statistically different (*p* > 0.05)

We first confirmed that PLX treatment leads to a decrease in the number of microglia in the cerebellum by performing immunohistochemistry using the antibody that recognizes the microglia-specific ionized calcium-binding adapter molecule 1 (Iba-1). As expected, the PLX treatment resulted in an 82% decrease in microglial density in the cerebella of wild-type mice and a 69% decrease in microglial density in the cerebella of SCA1 mice (one-way ANOVA with Bonferroni post-testing *p* < 0.05, Fig. 1a, b). However, PLX treatment lead to hypertrophy of microglia in wild-type and in *ATXN1[82Q]* mice (Fig. 1a insets). We have confirmed decreased Iba1 protein levels using Western-blotting (one-way ANOVA with Bonferroni post-testing, *p* < 0.05, Figs. 1e, f).

To complement these studies, we determined the expression of microglia specific genes (*Itgam* and *Aif1*) using quantitative reverse transcriptase polymerase chain reaction (qRT-PCR). Expression of both *Itgam* and *Aif1* was significantly decreased in cerebellar extracts of PLX-treated wild-type and SCA1 mice (one-way ANOVA with Bonferroni post-testing *p* < 0.05, Fig. 1c, d). These results further confirm that PLX treatment reduces microglia both in wild-type and SCA1 mice.

### PLX treatment improved motor behavior of *ATXN1[82Q]* mice

**Fig. 2:**
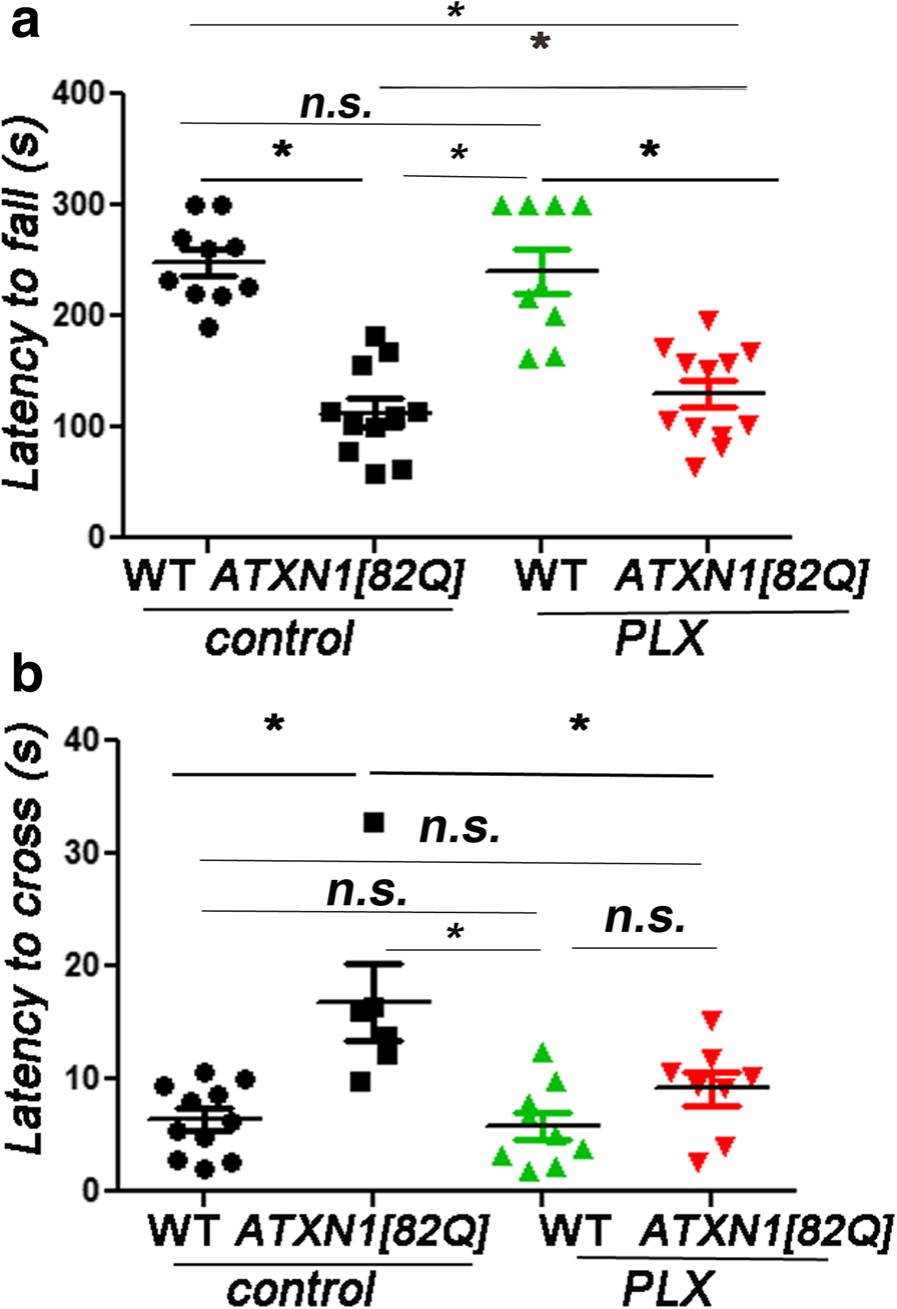
PLX treatment ameliorates motor deficits of SCA1 mice. After 2 months of PLX treatment, WT and *ATXN1[82Q]* and untreated controls were tested on a rotarod (**a**, *N* ≥ 10) and balance beam (**b**, *N* ≥ 6) at 3 months of age. Each dot represents one mouse, and values indicate mean ± SEM. *Asterisk* indicates *p* < 0.05 by one-way ANOVA followed by Bonferroni post hoc test and one-way ANOVA with pairwise permutation tests, using Benjamini-Hochberg procedure *p* value adjustment, presence of *n.s.* indicates that *p* > 0.05 and that data was not significantly different

One of the earliest symptoms of SCA1 in patients is ataxia, which refers to a loss of motor control and balance that can be best quantified in SCA1 transgenic mice by the accelerating rotating rod (rotarod) and balance beam tests [46]. In the rotarod test, mice that have cerebellar deficits tend to fall off the rotating rod early as it accelerates; the time it takes for a mouse to fall is recorded (latency to fall). In the balance beam test, the time required to cross a beam (latency to cross) is increased in mice with cerebellar dysfunction. We next examined whether PLX-induced reduction in microglial density during the early stages of SCA1 affected the onset and/or severity of motor deficits by using these tests. Motor behavior tests were performed at 3 months of age, which is at the onset of motor behavior deficits, and after 2 months of PLX treatment. Wild-type mice treated with PLX were undistinguishable from untreated wild-type mice both on rotarod and balance beam tests (Fig. 2a, b, one-way ANOVA *p* > 0.05), thus suggesting that loss of cerebellar microglia is well tolerated in wild-type mice and on its own does not cause motor deficits.

However, PLX-treated-*ATXN1[82Q]* mice demonstrated improved performance on the rotarod (latency means ± SEM were control WT mice 248 ± 11 s; PLX WT mice 240.9 ± 19 s; control *ATXN1[82Q]* mice 105.6 ± 13 s; and PLX *ATXN1[82Q]* mice 126.5 ± 12 s) and balance beam tests (latencies were control WT mice 6.42 ± 0.94 s, PLX WT mice 5.88 ± 1.2 s, control *ATXN1[82Q]* mice 16.88 ± 3.3 s, and PLX-treated *ATXN1[82Q]* mice 9.16 ± 1.44) compared to control-treated-*ATXN1[82Q]* mi*ce* (Fig. 2a, b, *p* < 0.05 one-way ANOVA with pairwise permutation tests using Benjamini-Hochberg procedure *p* value adjustment and one-way ANOVA with Bonferroni post-test), suggesting that microglial reduction ameliorates motor symptoms in SCA1 mice.

### PLX treatment decreased expression of pro-inflammatory cytokine TNFα

One way by which activated microglia are thought to interact with other cell types and contribute to neurodegeneration is through the secretion of pro-inflammatory cytokines [3, 9]. Therefore, we examined if PLX-mediated reduction in microglia alters expression of pro-inflammatory factor tumor necrosis factor alpha (TNFα), and monocyte chemoattractant protein-1 (MCP-1) previously shown to be increased early in SCA1 mice [4].

**Fig. 3:**
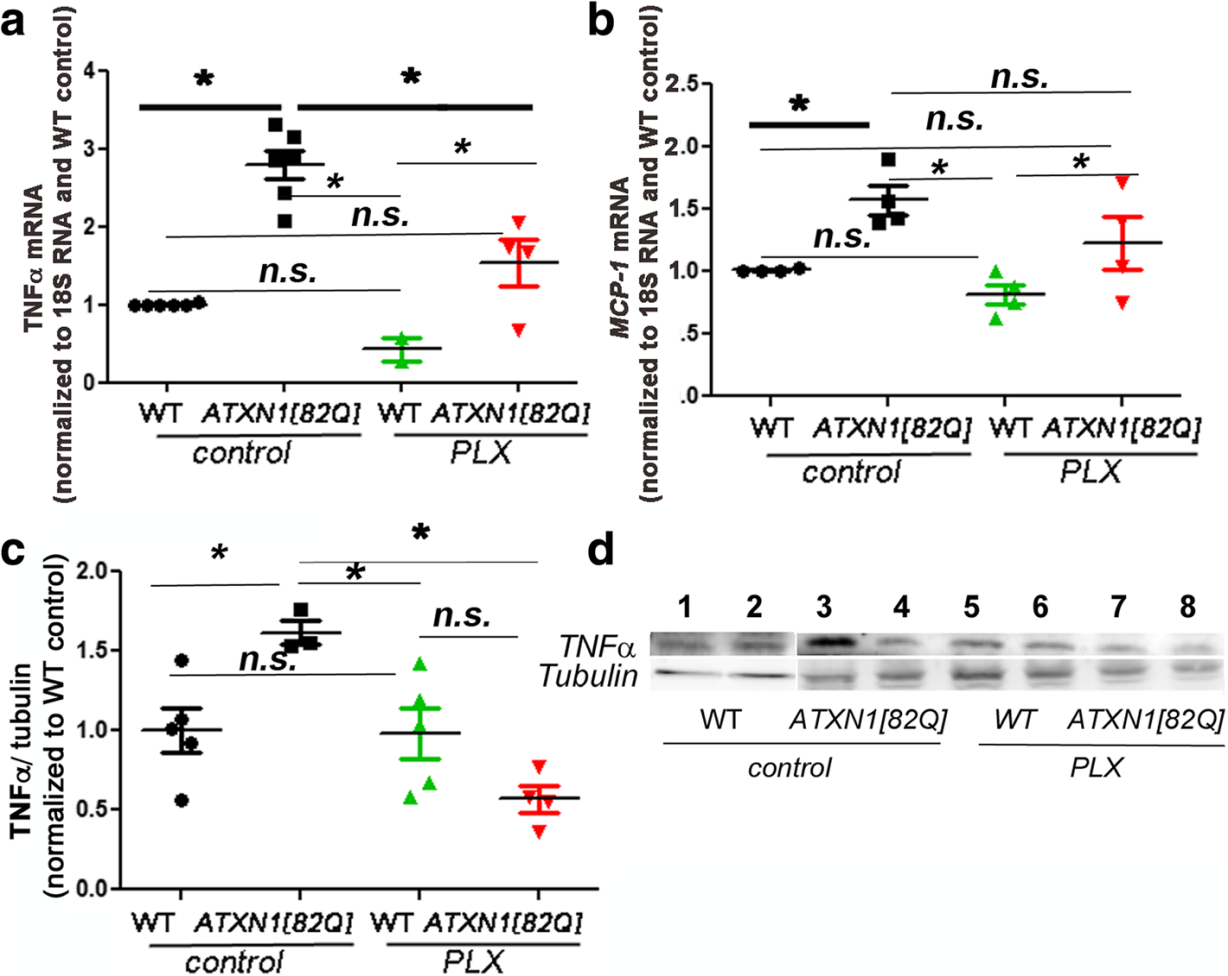
PLX treatment reduces expression of pro-inflammatory cytokine TNFα. RNA expression of *TNFα* (**a**) and *MCP-1*(**b**) in cerebellar samples from PLX and control SCA1 and wild-type mice with reference to control wild-type littermates and normalized to 18S rRNA. **c**, **d** Protein level of TNFα was quantified at 20 kD using Western blotting. *N* ≥ 3. Each dot represents one mouse, and values indicate mean ± SEM. *Asterisk* indicates *p* < 0.05 by one-way ANOVA followed by Bonferroni post hoc test, presence of *n.s.* indicates that *p* > 0.05 and that data was not significantly different and were not marked

As expected, *ATXN1[82Q]* mice showed an increased expression of TNFα and MCP-1 compared to WT (Fig. 3a, b, one-way ANOVA, *p* < 0.05) [4]. PLX treatment caused a significant decrease in the expression of TNFα at mRNA and protein levels in SCA1 mice (Fig. 3a, c, one-way ANOVA with Bonferroni post-testing, *p* < 0.05), and a trend towards a reduced expression of MCP-1 (Fig. 3b, one-way ANOVA, *p* > 0.05). Overall, these results suggest that PLX decreases expression of pro-inflammatory cytokine TNFα.

### Microglial depletion did not affect cerebellar astrogliosis in *ATXN1[82Q]* mice

**Fig. 4:**
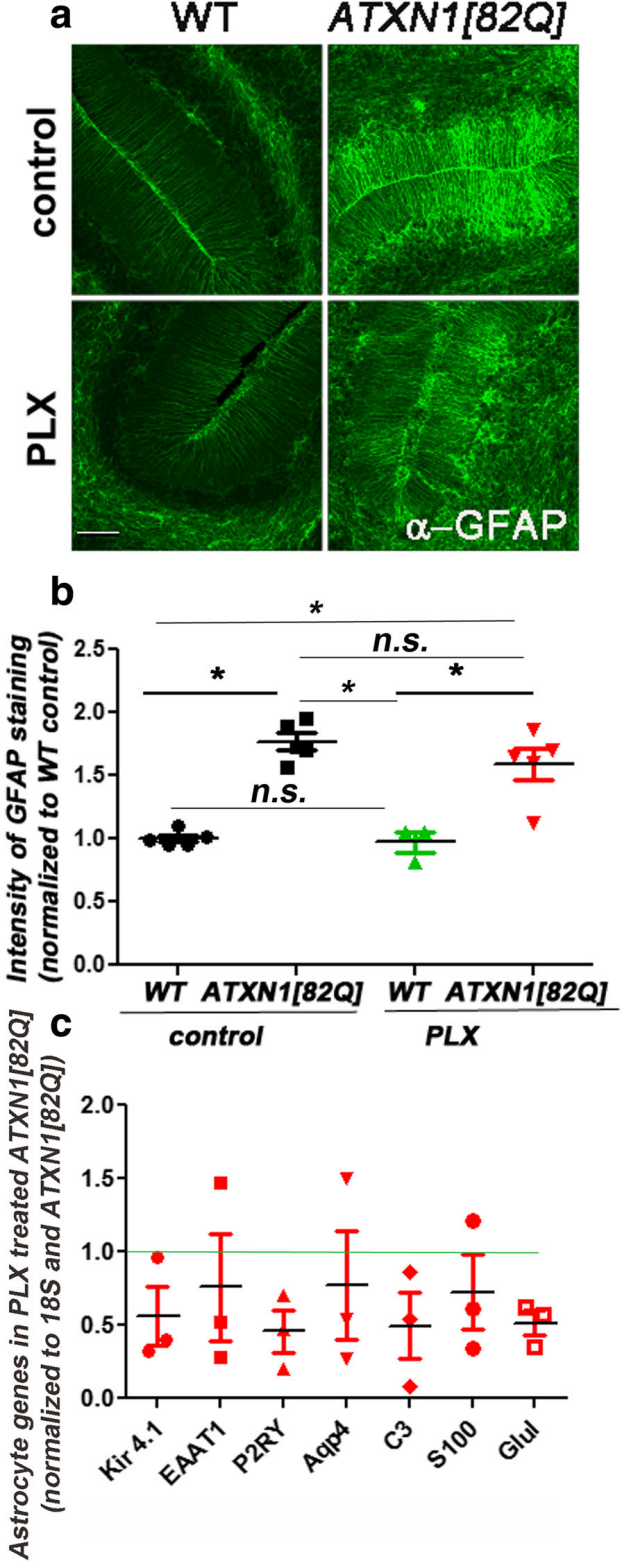
PLX does not alter cerebellar astrogliosis in SCA1 mice. **a** Representative confocal images of 3-month-old mice stained with an astrocyte-specific anti-GFAP antibody on the WT and *ATXN1[82Q]* mice fed regular (control) or PLX chow (PLX). *Scale bar*, 50 μm. **b** Quantification of GFAP intensity. **c** qRT-PCR was used to determine expression of astrocyte genes in cerebellar samples from PLX and control SCA1 and their wild-type littermate controls with reference to control WT mice (represented by *green line*) and normalized to 18S RNA. *N* ≥ 3. Each dot represents one mouse, and values indicate mean ± SEM. *Asterisk* indicates *p* < 0.05 by one-way ANOVA followed by Bonferroni post hoc test, presence of *n.s.* indicates that *p* > 0.05 and that data was not significantly different

Astrocytes are another CNS cell type important in neuroinflammation [48]. Similar to microglia, astrocytes become activated early in SCA1 and are closely associated with disease pathology. Activated microglia are thought to coordinate with astrocytes to drive neuroinflammatory signaling in many neurodegenerative conditions [8], and crosstalk between astrocytes and microglia has been proposed to play an important role in CNS diseases. Therefore, we next tested if microglial depletion early in SCA1 affects the activation of astrocytes [4]. Astrocyte activation is characterized by the hypertrophy of cell bodies and increased expression of glial fibrillary acidic protein (GFAP), an intermediate filament protein specific for astrocytes [48]. As we have previously shown, SCA1 mice showed an increased GFAP intensity (Figs. 4a, b, one-way ANOVA with Bonferroni post-testing, *p* < 0.05). Microglial depletion caused a slight, but not significant, decrease in GFAP intensity in wild-type mice, and PLX-treated *ATXN1[82Q]* mice showed a trend toward a decrease in GFAP expression (Fig. 4b, one-way ANOVA, *p* > 0.05). Similarly, expression of astrocyte genes, which are normally increased in *ATXN1[82Q]* mice (*Kir4.1, EAAT1, P2RY, Aqp4, C3, S100, Glul*), were reduced with PLX treatment. Yet, this trend did not reach statistical significance (Fig. 4c, one-way ANOVA, *p* > 0.05).

### Microglial depletion with PLX does not significantly alter pathology of Purkinje neurons in SCA1 mice

**Fig. 5:**
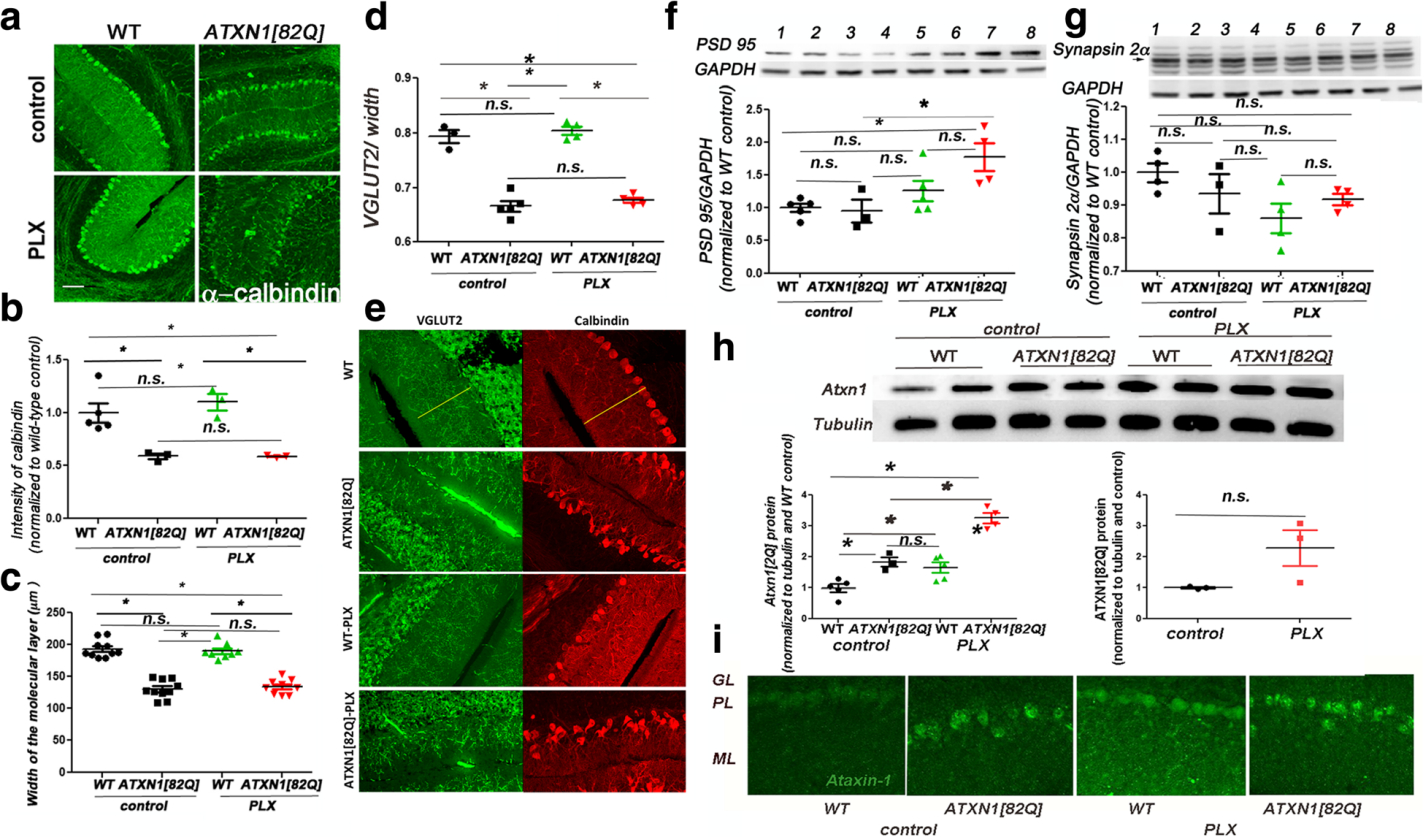
PLX does not improve pathological changes in Purkinje neurons. **a** Representative confocal images of 3-month-old WT and *ATXN1[82Q]* mice fed regular (control) or PLX chow (PLX). Cerebellar slices were stained with a Purkinje neuron specific anti-calbindin antibody. *Scale bar*, 100 μm. **b** Quantification of Calbindin intensity. **c** Width of the molecular layer. **d**, **e** To investigate synaptic loss, cerebellar slices were co-stained with VGLUT-2 and calbindin. Quantification of the extension of VGLUT2 positive synaptic puncta over the width of molecular layer (**d**). Note that in these images, decrease in calbindin is not detectable (**e**). The reason for this is that for imaging calbindin in this experiment, we have used different exposures to obtain similar calbindin intensity over samples to increase accuracy of quantifying the length of VGLUT2 staining over calbindin. **f**, **g** WB was used to quantify protein expression of pre-synaptic marker synapsin 2a (**g**, indicated by *arrow*) and post-synaptic marker PSD95 (**f**) *N* ≥ 3 . In representative WB image, samples are wild-type controls (lanes 1 and 2), *ATXN1[82Q]* controls (lanes 3 and 4), wild-type PLX-treated mice (5 and 6), and *ATXN1[82Q]* PLX-treated mice (7 and 8). **h**, **i**. Ataxin-1 protein levels were examined using Western blotting of cerebellar lysates from 3-month-old PLX and control-treated SCA1 and wild-type mice (**h**) and immunofluorescence of cerebellar slices (**i**). N ≥ 3. Each dot represents one mouse, and values indicate mean ± SEM. *Asterisk* indicates *p* < 0.05 by one-way ANOVA followed by Bonferroni post hoc test or Student’s *t* test, presence of *n.s.* indicates that *p* > 0.05 and that data was not significantly different

We next examined whether the reduced neuropathology underlies the ameliorated motor deficits in SCA1 mice after PLX treatment. Because SCA1 neurodegeneration is most pronounced in the cerebellar Purkinje neurons (PN), we focused on evaluating cerebellar histopathology. We stained PNs and their neurites with a calbindin-D-28K antibody to document alterations in dendritic arborization, which is the earliest sign of SCA1 pathogenesis [49]. As expected, we found that calbindin staining intensity was significantly reduced in SCA1 mice compared with wild-type controls (Figs. 5a, b, one-way ANOVA with Bonferroni post-test, *p* < 0.05). We have also found that PLX treatment did not significantly alter calbindin intensity in wild-type mice (Fig. 5b, one-way ANOVA with Bonferroni post-test, *p* > 0.05). However, we did not observe any significant improvement in calbindin intensity after PLX treatment in SCA1 mice (Fig. 5b, one-way ANOVA with Bonferroni post-test, *p* > 0.05). In addition to the decreased intensity of calbindin staining, dendritic pathology in SCA1 mice can be quantified as a decrease in the width of the molecular layer and loss of climbing fiber synapses (characterized by vesicular glutamate transporter 2 (VGLUT2) onto Purkinje neurons) [50, 51]. As previously shown, *ATXN1[82Q]* mice showed a significantly reduced molecular layer width and length of VGLUT2 reactivity [40], but they were not rescued by PLX treatment (Figs. 5c–e, one-way ANOVA with Bonferroni post-test, *p* > 0.05). To further examine the synaptic changes, we tested for the expression of pre-synaptic and post-synaptic protein synapsin and post-synaptic density 95 (PSD95). We found that PLX treatment significantly increased expression of PSD95 in *ATXN1[82Q]* mice (Fig. 5f, one-way ANOVA with Bonferroni post-test, *p* < 0.05) and have detected no significant changes in the expression of pre-synaptic marker synapsin (Fig. 5g, one-way ANOVA with Bonferroni post-test, *p* > 0.05).

Recent studies have identified a Purkinje neuron gene module whose expression correlates with onset and progression of SCA1 [46]. We have examined whether PLX treatment alters the expression of the subset of these genes in Purkinje neurons (*calbindin, ITPR, INPP5a, Garn13, PCP4, Homer3, and Rgs8*). However, we have found only a trend towards rescue of some of these genes (*ITPR, INNPP5a, Garn13, PCP4 and Rgs8*) in PLX-treated SCA1 mice (Additional file 1: Figure S1, one-way ANOVA with Bonferroni post-test, *p* > 0.05).

Expansion of polyQ repeats increases misfolding and reduces ataxin-1 clearance leading to the accumulation of both soluble and aggregated ataxin-1 [52]. Therefore, we next tested whether PLX treatment affects protein levels of ataxin-1 [53]. There was no statistically significant change in the protein levels of mutant soluble ATXN1[82Q] (Fig. 5h right, Student’s *t* test with Welch’s correction *p* = 0.124) nor in the number of ATXN1[82Q] aggregates in SCA1 mice with PLX treatment (data not shown, *N* = 5, Student’s *t* test *p* = 0.63). Surprisingly, we have found that PLX treatment *increased* wild-type Atxn1[2Q] levels in wild-type and SCA1 mice (Fig. 5h left, one-way ANOVA with Bonferroni post-test, *p* < 0.05). Immunohistochemistry demonstrated that this increase in ataxin-1 protein is in the cerebellar Purkinje neurons (Fig. 5i). Overall, these data suggest that PLX treatment increases protein levels of wild-type ataxin-1, but does not significantly alter mutant ataxin-1.

## Discussion

We have designed this study to test the role of the microglia during the early stages of disease in the transgenic mouse model of SCA1. Our data demonstrated that depleting microglia during the early stage improved motor deficits in SCA1 mice, suggesting that microglia may play an important role in the pathogenesis of SCA1. However, we did not detect a significant change in PN atrophy and synaptic loss treatment nor significantly altered astrogliosis, including changes in GFAP expression and rescue of gene expression changes in reactive astrocytes in SCA1 mice treated with PLX. These data may suggest that dysfunction of Purkinje neurons is sufficient to activate astrocytes in SCA1, or that remaining microglia can still cause astrogliosis, and other cerebellar glia, such as Bergman glia, may be able to remove synapses in absence of microglia.

We further investigated the molecular mechanisms underlying the ameliorated motor function, and we found that increased expression of PSD95 and ATXN1[2Q], and reduced pro-inflammatory cytokine TNFα expression with PLX treatment may be beneficial to alleviate pathogenesis of SCA1. Despite not finding changes in VGLUT2 climbing fiber synapses in PLX-treated SCA1 mice, the increase in the post-synaptic marker PSD95 may indicate a homeostatic mechanism to preserve the functions of remaining synapses. In addition, our data also showed that depleting microglia decreased expression of pro-inflammatory cytokine TNFα. This result suggests that, at least in *ATXN1[82Q]* mice, microglia produce the majority of TNFα, and that this decrease in TNFα in PLX-treated *ATXN1[82Q]* mice may contribute to improved motor function [54]. Intriguingly, PLX increased the level of wild-type Atxn1[2Q]. Previous studies have demonstrated that wild-type ataxin-1 is protective in SCA1. It has been demonstrated that polyQ mutation leads to both loss and gain of toxic function, and that reducing levels of wild-type ataxin-1 worsens SCA1 disease phenotype. Therefore, this increase in wild-type ataxin-1 may be beneficial and promote function of Purkinje neurons [52]. While the underlying mechanism by which PLX treatment results in increased ataxin-1 expression is unclear, it is possible that PLX may directly regulate ataxin-1 through c-kit and Flt3 signaling (also inhibited by PLX), or that microglia and neuroinflammation may modulate protein clearance in neurons in neurodegenerative disease.

Increasing evidence suggests that glial cells, including microglia, astrocytes, and oligodendrocytes, are critical for the functions of CNS, yet their roles in diseases and interactions with each other remain controversial. In many neurodegenerative diseases, including SCA1, microglia undergo morphological and functional changes, including enlargement of their cell bodies, thickening of their processes, and increased expression of pro-inflammatory cytokines [55–57]. In this study, we have used the transgenic mouse model of SCA1 in which mutant protein is expressed only in Purkinje neurons. In these mice, microglia are activated in response to neuronal dysfunction only 1 week after the onset of the mutant ATXN1[82Q] expression in neurons. Such early activation of microglia in *ATXN1[82Q]* mice demonstrates that cerebellar microglia are closely tuned to neuronal dysfunction [4]. We have previously demonstrated that cerebellar astrocytes are also activated early in SCA1. Astroglia are critical for neuronal functions under non-pathological conditions, and Bergman glia (BG), a type of cerebellar astrocyte that interacts very closely with Purkinje neurons, perform many important physiological functions including regulation of ion homeostasis, uptake of released neurotransmitters, secretion of neurotrophic factors, and synaptic remodeling. In disease, astrocytes also undergo activation that results in morphological and functional changes, including enlarged cell bodies, thickening of cell processes, altered ability to maintain homeostasis of ions and neurotransmitters, and production of neurotrophic factors. Early in disease progression, astrocytes in SCA1 mice are enlarged and have increased expression of GFAP and astrocytic genes that regulate homeostasis and promote neuronal survival, such as Kir4.1. Microglia have the capacity to interact with astrocytes and thus modulate their activation. Because both astrocytes and microglia are activated around the same time in SCA1, it is possible that microglia contribute to activation of astrocytes. Since depletion of microglia did not significantly alter hallmark signs of astrogliosis, increase in GFAP expression, and only a trend towards change in astroglial gene expression, this result may indicate that either microglia do not modulate astroglial activation, or that remaining microglia are sufficient to modulate astrogliosis. Oligodendrocytes are myelinating cells of the CNS that also contribute to trophic support of neurons. While oligodendrocytes are critical during cerebellar development, and increasing evidence suggests that both oligodendrocyte precursor cells and oligodendrocytes can contribute to neuroinflammation and disease progression in many diseases [58–61], very little is known about their role in SCA1. Future studies will explore whether oligodendrocytes are involved in SCA1. This is the first study to demonstrate that microglia contribute to the pathogenesis during the early stages of SCA1 by showing an amelioration of motor deficits when microglia are reduced early in disease. These results challenge the dogma that SCA1 is only a neuronal disease and provide evidence for the possibility of glia-based therapies for SCA1. We are intrigued by the observed increase in ataxin1 protein levels in Purkinje neurons after PLX treatment. While the underlying mechanism is not clear, this result may suggest that either normal function of microglia is to help regulate protein degradation in neurons or PLX directly modulates protein degradation in PN, acting through c-kit or Flt3. Identifying the molecular mechanism of this effect of PLX could provide novel therapies to prevent build up and aggregation of toxic proteins by enhancing protein degradation in neurons in SCA1 and other neurodegenerative diseases. It is important to note that microglial contribution to the pathogenesis of SCA1 may change with disease progression [5, 55]. Future studies testing the role of microglia in late stages of SCA1 could address this question.

## Conclusions

We have found that PLX reduced the number of microglia and microglial gene expression, including expression of pro-inflammatory cytokine TNFα in the cerebella of wild-type and SCA1 mice. Importantly, PLX significantly improved the motor performance of SCA1 mice, suggesting a potential therapeutic benefit. PLX did not significantly alter astrogliosis, but showed trend towards modulation of astroglial gene expression. PLX treatment increased protein levels of wild-type ataxin-1, indicating a possible role of microglia and/or c-kit and Flt3 signaling in neuronal protein clearance.

## Additional file

Additional file 1: Figure S1. Disease-associated gene expression changes in Purkinje neurons are not altered with PLX treatment. RNA expression of genes belonging to Magenta cluster in cerebellar samples from PLX and control SCA1 mice (with reference to control-treated wild-type and *ATXN1[82Q]* littermates and normalized to 18S RNA). *N* ≥ 3. Each dot represents one mouse, and values indicate mean ± SEM. (TIF 9227 kb)

## Abbrevations

AD: Alzheimer disease
ALS: Amyotrophic lateral sclerosis
ATXN1: Ataxin1
CSF1R: Colony-stimulating factor 1 receptor
GFAP: Glial fibrilary acidic protein
HD: Huntington disease
Iba1: Ionized calcium-binding adapter molecule 1
MCP-1: Monocyte chemoattractant protein-1
PD: Parkinson disease
PN: Purkinje neuron
SCA1: Spinocerebellar ataxia type1
TNFα: Tumor necrosis factor alpha
