# Supplementary figures and images for: Inhibition of colony-stimulating factor 1 receptor early in disease ameliorates motor deficits in SCA1 mice
Source: J Neuroinflammation. 2017 May 25;14:107. doi: 10.1186/s12974-017-0880-z (PMC5445366; doi:10.1186/s12974-017-0880-z)

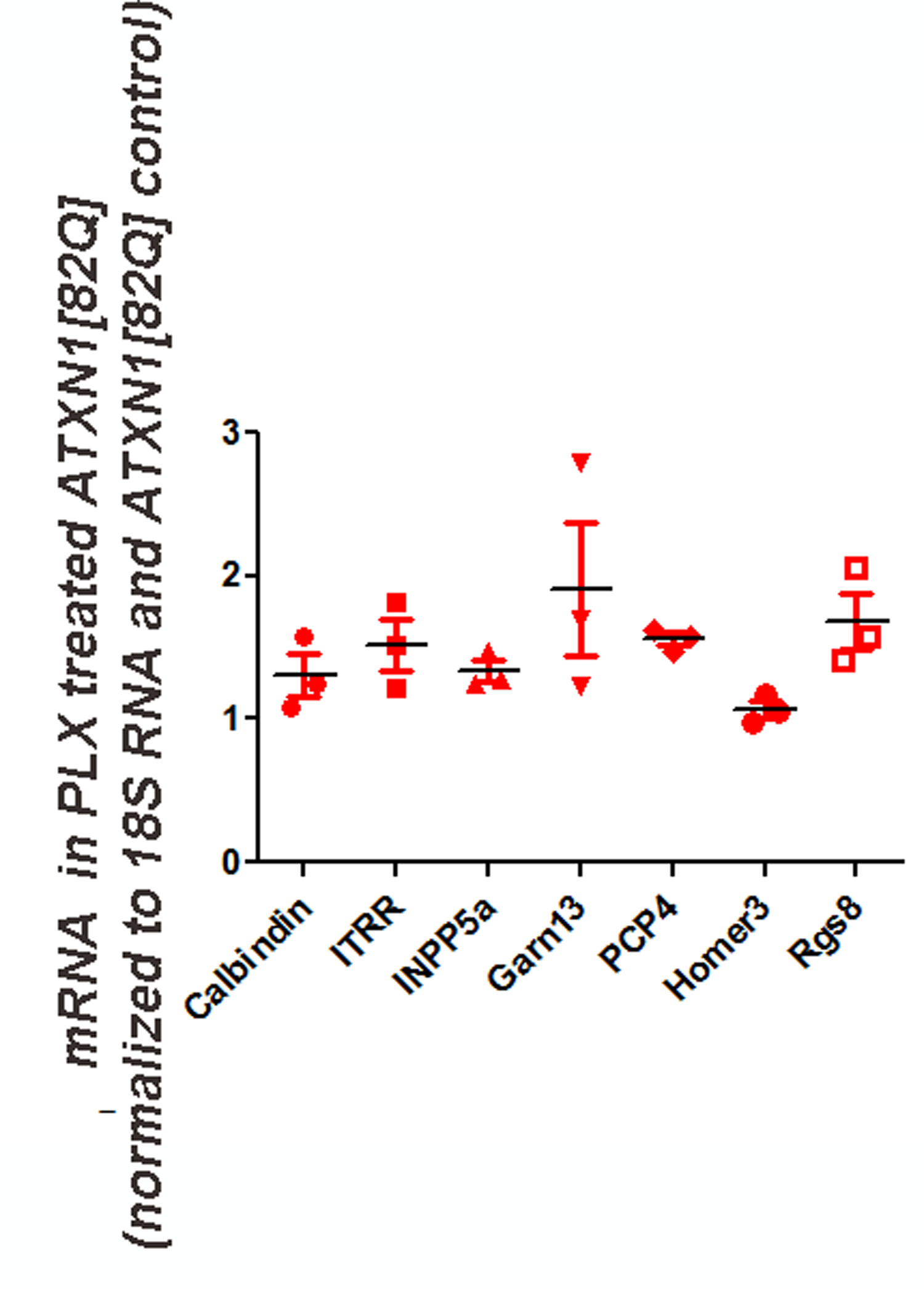

Supplement: Additional file 1: Figure S1. — Disease-associated gene expression changes in Purkinje neurons are not altered with PLX treatment. RNA expression of genes belonging to Magenta cluster in cerebellar samples from PLX and control SCA1 mice (with reference to control-treated wild-type and ATXN1[82Q] littermates and normalized to 18S RNA). N ≥ 3. Each dot represents one mouse, and values indicate mean ± SEM. (TIF 9227 kb) [file 12974_2017_880_MOESM1_ESM.tif]
